# Frequency and phenotype of GAA-*FGF14* disease in bilateral vestibulopathy syndromes: insights from repeat expansion carriers, including a case of co-occurrence with *RFC1*-related CANVAS

**DOI:** 10.1007/s00415-026-13867-1

**Published:** 2026-05-25

**Authors:** David Pellerin, Felix Heindl, Andreas Traschütz, Pablo Iruzubieta, Marie-Josée Dicaire, Stephan Zuchner, Annette M. Hartmann, Dan Rujescu, Henry Houlden, Bernard Brais, Michael Strupp, Matthis Synofzik

**Affiliations:** 1 Department of Neurology and Neurosurgery, Montreal Neurological Hospital and Institute, McGill University, Montreal, QC Canada; 2 Department of Neuromuscular Diseases, UCL Queen Square Institute of Neurology and The National Hospital for Neurology and Neurosurgery, University College London, London, UK; 3 Dr. John T. Macdonald Foundation Department of Human Genetics and John P. Hussman Institute for Human Genomics, University of Miami Miller School of Medicine, Miami, FL USA; 4 Department of Neurology and German Center for Vertigo and Balance Disorders, LMU University Hospital, LMU Munich, Germany; 5 Division Translational Genomics of Neurodegenerative Diseases, Hertie-Institute for Clinical Brain Research and Center of Neurology, University of Tübingen, Tübingen, Germany; 6 German Center for Neurodegenerative Diseases (DZNE), Tübingen, Germany; 7 Department of Neurology and Epileptology, Hertie-Institute for Clinical Brain Research and Center of Neurology, University of Tübingen, Tübingen, Germany; 8 Department of Neurosciences, Biogipuzkoa Health Research Institute, Donostia-San Sebastián, Spain; 9 CIBERNED Centro de Investigación Biomédica en Red en Enfermedades Neurodegenerativas, Instituto de Salud Carlos III (CIBER-CIBERNED-ISCIII), 28029 Madrid, Spain; 10 Department of Psychiatry and Psychotherapy, Comprehensive Center for Clinical Neurosciences and Mental Health (C3NMH), Medical University of Vienna, Vienna, Austria; 11 Department of Human Genetics, McGill University, Montreal, QC Canada

**Keywords:** *FGF14*, SCA27B, GAA-FGF14 ataxia, CANVAS, Cerebellar ataxia, Bilateral vestibulopathy

## Abstract

**Objectives:**

Intronic *FGF14* GAA repeat expansions cause spinocerebellar ataxia 27B (SCA27B) / GAA-*FGF14* disease. Bilateral vestibulopathy (BVP) has been reported as a recurrent feature of this disease. Here, we aimed to determine whether GAA-*FGF14* expansions represent a common cause of primary BVP syndromes.

**Methods:**

*FGF14* genotyping, and in-depth neurological, vestibular and disease evolution phenotyping of 116 consecutive patients meeting the diagnostic criteria for BVP, including 92 with idiopathic BVP, 10 with biallelic *RFC1* expansions, and 14 with a secondary cause.

**Results:**

Two patients in the idiopathic BVP group (2/92, 2.2%; 430 and 349 GAA repeats) and one in the *RFC1*-positive BVP group (1/10, 10%; 255 GAA repeats) carried an *FGF14* (GAA)_≥250_ expansion. No expansions were detected in the secondary BVP group. In the idiopathic BVP group, the GAA-*FGF14*-positive patients had mild-to-moderate BVP and a phenotype that aligned with SCA27B. The patient carrying both *FGF14* and biallelic *RFC1* expansions developed cerebellar dysfunction, downbeat nystagmus, sensory neuropathy, and BVP at age 70. Downbeat nystagmus was observed in all GAA-*FGF14* expansion carriers (3/3, 100%) compared to only a minority of patients with idiopathic BVP (7/90, 8%; Fisher’s exact test, *p* = 0.0009).

**Discussion:**

The phenotypic spectrum of GAA-*FGF14* disease can include a relevant bilateral vestibular deficit (BVP); however, *FGF14* GAA expansions are overall a rare cause of primary BVP syndromes. Given the possible co-occurrence of GAA-*FGF14* and *RFC1* expansions, dual diagnosis should be considered in patients presenting with unusual or broader phenotypes.

## Introduction

Bilateral vestibulopathy (BVP) is a chronic disorder characterized by bilaterally reduced or absent function of the peripheral and/or, rarely, the central vestibular system, leading to unsteadiness during standing and walking that worsens in darkness or on uneven ground, as well as movement-induced oscillopsia [1, 2]. Multiple etiologies can lead to BVP, the most common of which include ototoxic drugs, infectious causes, Ménière’s disease, and genetic disorders [1–4]. Notably, a recent cohort study identified biallelic *RFC1* repeat expansions – known to cause cerebellar ataxia, neuropathy, and vestibular areflexia syndrome (CANVAS) [5] – in 8% of previously idiopathic BVP cases [6], suggesting that additional monogenic causes may underlie a proportion of cases that remain unexplained. Nonetheless, despite these known causes, BVP is still considered idiopathic in up to 20–50% of patients [1–4].

Dominantly inherited GAA repeat expansions in the first intron of *FGF14* have recently been identified as the cause of spinocerebellar ataxia 27B (SCA27B)/GAA-*FGF14* disease [7, 8], a late-onset, slowly progressive cerebellar ataxia that is commonly associated with episodic symptoms and downbeat nystagmus [9–11]. Vestibular hypofunction has also been reported as a recurrent feature of GAA-*FGF14* disease due to cerebellar flocculo-nodular involvement, with some series suggesting a frequency as high as 70% [10, 12–15]. Furthermore, when present, BVP appears to remain relatively mild and be a late feature in GAA-*FGF14* disease compared to cerebellar ataxia, as it was shown to develop on average more than 10 years after disease onset [10, 14]. However, *FGF14* repeat expansions have not yet been systematically screened in patients with a primary presentation of BVP, and it remains unknown whether such expansions represent a common cause of primary BVP syndromes.

Here, we hypothesized that *FGF14* GAA repeat expansions may represent a monogenic cause of BVP and that BVP may be part of the phenotypic spectrum of GAA-*FGF14* disease. To investigate this, we screened for *FGF14* repeat expansions in a large consecutive cohort of patients with BVP and delineated the phenotypic characteristics of GAA-*FGF14*-related BVP.

### Methods

**Fig. 1:**
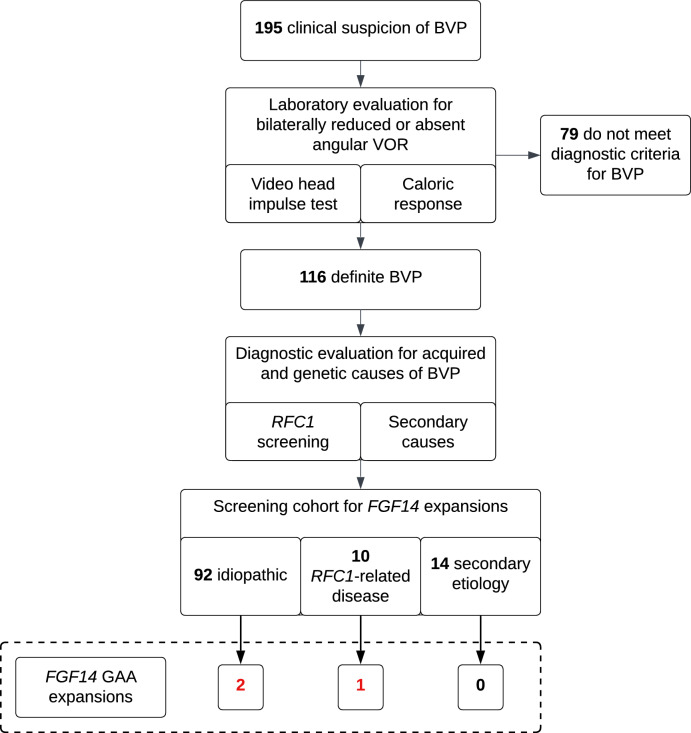
Study flowchart of the recruitment of patients with BVP. *BVP* bilateral vestibulopathy; *VOR* vestibulo-ocular reflex

We retrospectively enrolled a consecutive series of 195 index patients with a clinical suspicion of BVP referred to the Department of Neurology and the German Center for Vertigo and Balance Disorders at the LMU Hospital in Munich, Germany. Some patients in this cohort (*n* = 168) have previously been reported [6]. Following referral, patients underwent laboratory evaluation of vestibular function by video head impulse test (vHIT; *n* = 192) and/or caloric stimulation (*n* = 185) to confirm bilateral reduction or absence of angular vestibulo-ocular reflex (VOR) function, in accordance with the Bárány Society diagnostic criteria (horizontal angular VOR gain < 0.6 on vHIT and/or reduced caloric response) [1]. Patients were excluded from this study if they did not meet diagnostic criteria for BVP (*n* = 79). The final study cohort comprised 116 patients meeting diagnostic criteria for BVP, which requires objective evidence based on quantitative vestibular testing (Fig. 1) [1]. All but three patients were of self-reported European ancestry. Patients underwent thorough clinical assessment in the vestibular center of one of the authors with leading expertise in vestibular and cerebellar disorders (M. St.), as well as comprehensive etiologic evaluation that included screening for *RFC1* repeat expansions and assessment for acquired causes through detailed medical history, review of drug exposure, neurological and VOR examination, and laboratory testing. Genetic testing for ataxia was performed in select patients when deemed appropriate by the clinician. Screening for *RFC1* AAGGG expansions was performed as described previously [5, 6]. Following diagnostic evaluation, 10 patients were found to carry biallelic pathogenic *RFC1* repeat expansions, 14 had a secondary acquired cause of BVP, and 92 had no identified etiology and were therefore classified as “idiopathic” BVP (Fig. 1).

All 116 patients with definite BVP, regardless of the underlying cause, underwent genetic testing for *FGF14* repeat expansions, as described previously [16]. Patients with *RFC1*-related BVP and secondary causes of BVP were not excluded from *FGF14* screening to explore the possibility of co-occurring/dual diseases. Expansions of ≥ 250 GAA-pure repeat units were considered pathogenic [17].

Deep phenotyping was performed by systematically reassessing all medical records using a standardized datasheet while being blind to the underlying genotype (GAA-*FGF14*, *RFC1*). Cerebellar impairment was diagnosed upon documentation of cerebellar ocular motor signs (e.g., downbeat nystagmus, dysmetric saccades, gaze-evoked nystagmus, etc.) and/or cerebellar dysarthria and/or cerebellar ataxia defined by the presence of dysdiadochokinesia, intention tremor, or ataxia of the upper limbs. Polyneuropathy was diagnosed on nerve conduction studies (NCS), if performed, or clinically defined by the combination of decreased vibration sense at the ankles (≤ 3/8 on the Rydel-Seiffer scale) and decreased ankle reflexes [18]. Results of brain MRI and NCS were available for review in 75 and 14 patients, respectively.

This study was approved by the ethics committee of the LMU University Hospital, LMU Munich, Germany (IRB# 379–11) and we obtained written informed consent from all the participants.

## Results

### Frequency of *FGF14* GAA repeat expansions in BVP syndromes

**Fig. 2:**
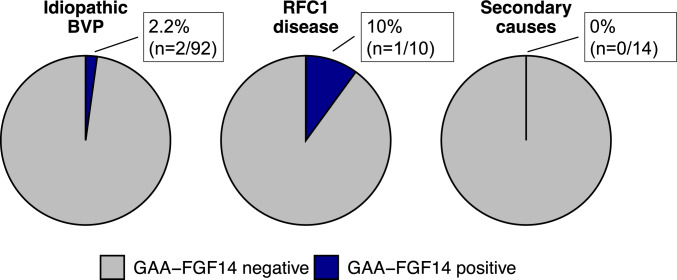
Frequency of *FGF14* GAA repeat expansions in BVP syndromes. Percentage of patients carrying an *FGF14* GAA repeat expansion among those with “idiopathic” BVP, *RFC1*-related BVP, and BVP attributed to a secondary acquired cause

An *FGF14* (GAA)_≥250_ repeat expansion was identified in 3 of 116 patients with definite BVP (2.6% of the entire study cohort; Fig. 1). This included 2 of 92 patients with “idiopathic” BVP (2.2%; 430 and 349 GAA repeat units) and 1 of 10 patients with *RFC1*-related BVP (10%; 255 GAA repeat units) (Fig. 2**)**. None of the 14 patients with a secondary, acquired cause of BVP carried a GAA-*FGF14* expansion. These secondary causes included previous exposure to ototoxic drug, Ménière disease, infections, and autoimmune diseases. Additionally, two patients with “idiopathic” BVP carried a GAAGGA expansion in *FGF14*, which is considered non-pathogenic [19].

**Fig. 3:**
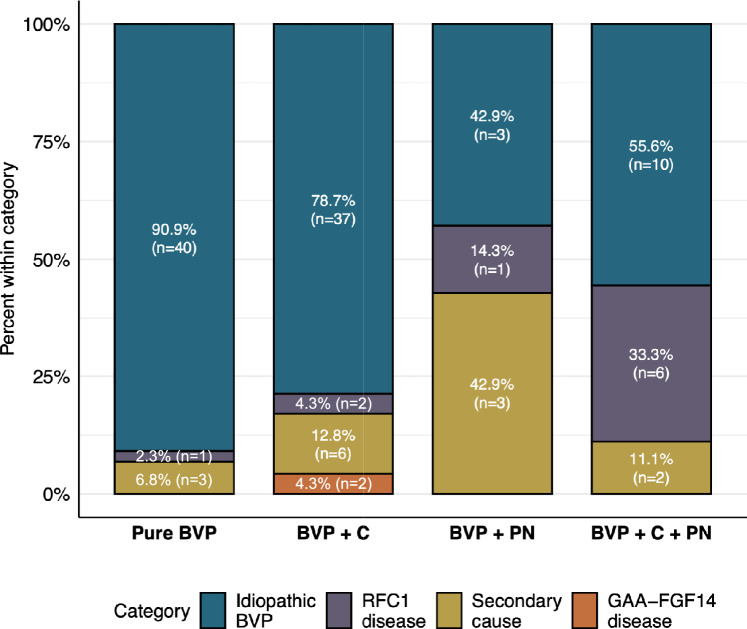
Etiological profiles across BVP subgroups. Distribution of “idiopathic”, *RFC1*-related, secondary, and GAA-*FGF14*-related cases among patients with pure/isolated BVP, BVP with cerebellar dysfunction (BVP + C), BVP with polyneuropathy (BVP + PN), and BVP combined with cerebellar dysfunction and polyneuropathy (BVP + C + PN). The *RFC1*-positive patient carrying an *FGF14* expansion was excluded from the GAA-*FGF14* category because the penetrance and phenotypic contribution of this expansion are unclear

When dividing the cohort according to four phenotypic groups-pure/isolated BVP, BVP with cerebellar dysfunction (BVP + C), BVP with polyneuropathy (BVP + PN), and BVP with both cerebellar dysfunction and polyneuropathy (BVP + C + PN)-both patients carrying an *FGF14* repeat expansion fell within the BVP + C group. The patient harboring both *RFC1* and *FGF14* repeat expansions was classified as BVP + C + PN, consistent with the clinical spectrum of CANVAS (Fig. 3). Notably, no *FGF14* expansion carriers were observed in the pure BVP or BVP + PN groups. The median (range) repeat size of the largest allele within each subgroup was 38.5 (7–344, the largest allele being a non-pathogenic GAAGGA expansion) for pure/isolated BVP, 48 (8–430) for BVP + C, 20 (15–63) for BVP + PN, and 46 (8–255) for BVP + C + PN.

Emerging evidence suggests that expansions of 200–249 GAA repeats may be pathogenic in a subset of individuals, albeit with incomplete penetrance [10, 17, 20, 21]. We identified four patients with “idiopathic” BVP carrying such alleles (200, 212, 222, and 235 repeats). While one patient exhibited saccadic pursuit and polyneuropathy, none had downbeat nystagmus (DBN) or cerebellar ataxia on examination, precluding a definitive conclusion regarding the pathogenicity of these alleles. No patient with secondary or *RFC1*-related BVP carried a 200–249 repeat allele.

### Phenotypic profile of the GAA*-FGF14*-positive patients with BVP

Table 1 summarizes the clinical features of the GAA-*FGF14*-positive patients. Patients previously classified as having “idiopathic” BVP who were found to carry an *FGF14* GAA repeat expansion developed cerebellar dysfunction, DBN, and BVP at ages 55 and 62, without episodic symptoms at onset. They carried an *FGF14* (GAA)_430_ and (GAA)_349_ expansion, respectively. None reported a family history of ataxia or BVP. Their first symptom was gait imbalance. They did not require walking aids, but both experienced recurrent falls. At their last evaluations, at ages 85 and 65 years – corresponding to disease durations of 30 and 3 years, respectively – they exhibited additional cerebellar ocular motor signs, including horizontal gaze-evoked nystagmus, rebound nystagmus, and saccadic pursuit. The patient with 30 years of disease duration also had cerebellar dysarthria and appendicular and gait ataxia . Neither showed clinical evidence of polyneuropathy (based on preserved vibration sense and lower-extremity reflexes). At their last examination, both patients had a Friedreich Ataxia Rating Scale (FARS) functional stage of 3, indicating mild disability, despite substantially different disease durations. Brain MRI performed at ages 74 and 64 years revealed no cerebellar atrophy. Their mean VOR gains on video head impulse test were 0.22° and 0.59°, consistent with mild-to-moderate BVP. Overall, the phenotype of both patients was consistent with the cerebellar-predominant pattern typical of SCA27B, while also exhibiting a relevant vestibular phenotype **(**Fig. 3). 4-aminopyridine was prescribed to the first patient at age 74, but discontinued a week later due to a prolonged QTc interval.

**Table 1:** Clinical characteristics of GAA-*FGF14*-positive patients

	Patient #16	Patient #17	Patient #116
Sex	Male	Female	Male
*FGF14* repeat genotype	46/430 GAA	8/349 GAA	10/255 GAA
Biallelic *RFC1* AAGGG expansions	−	−	+
Family history	−	−	−
Episodic ataxia	−	−	−
Age at onset	55	62	70
Disease duration	19	3	2
Walking aid	−	−	−
FARS disability stage (age)	2.5 (74); 3 (85)	3 (65)	2 (70); 2 (72)
Gait ataxia	+	+	+
Appendicular ataxia	+	−	unknown
Cerebellar dysarthria	+	−	unknown
Downbeat nystagmus	+	+	+
Gaze-evoked nystagmus	+	+	+
Polyneuropathy	−	−	+
Cerebellar atrophy	−	−	N/A
Average VOR gain	0.22°	0.59°	0.34°

*FARS* Friedreich Ataxia Rating Scale; *VOR* vestibulo-ocular reflex

The GAA–*FGF14*–positive patient in the *RFC1*-positive group developed cerebellar dysfunction, sensory neuropathy, and BVP at age 70 years, consistent with CANVAS. He carried an *FGF14* (GAA)_255_ expansion. He initially presented with gait impairment. He reported no family history of ataxia or BVP. After 2 years of disease progression, he exhibited gait ataxia, DBN, and saccadic pursuit, but still reported no episodic symptoms. He remained minimally disabled with a FARS functional stage of 2 at his last assessment at age 72 years, and did not require a walking aid. No brain MRI was available for review. The mean VOR gain on video head impulse test was 0.34°. Although this patient had a phenotype clearly consistent with CANVAS and carried an *FGF14* expansion, it is unclear whether the latter contributed to his phenotype. However, the presence of DBN could be related to SCA27B, as it appears to be more common in this condition (50–67%[9, 20, 22]) compared to *RFC1*-related disease (30%[6]).

### Discriminative features of the GAA-*FGF14*-related BVP syndrome

We next compared clinical features among patients with “idiopathic” (*n* = 90), *RFC1*-related (*n* = 10), secondary (*n* = 14), and GAA-*FGF14*-related (*n* = 2) BVP to identify features distinguishing the latter group (Table 2). For this analysis, the patient with *RFC1*-related BVP carrying a (GAA)_255_ *FGF14* expansion was kept in the *RFC1*-related group, as there was no unequivocal evidence of penetrance or phenotypic contribution from the *FGF14* expansion. Of note, however, DBN was observed in all GAA-*FGF14* expansion carriers (3/3, 100%) – here also including the individual in the *RFC1*-related BVP subgroup who exhibited DBN, a core feature of GAA-*FGF14* disease [9, 10] – compared to only a minority of patients with idiopathic BVP (7/90, 8%; Fisher’s exact test, *p* = 0.0009).

**Table 2:** Characteristics and discriminative features of the GAA*-FGF14*-related BVP syndrome

	**BVP**	**RFC1**	**Secondary**	**GAA-***FGF14*	**p value**	**q value**
No	90	10	14	2		
Male sex	60 (67%)	4 (40%)	9 64%)	1 (50%)	0.369	0.477
Repeat size of largest allele	41 (7–344)^a^	46 (16–255)	19 (8–108)	390 (349–430)		
Family history	12 (13%)	0 (0%)	0 (0%)	0 (0%)	0.417	0.500
Age at last exam	66.5 (22–88)	68 (39–78)	56.5 (21–81)	69.5 (65–74)	0.419	0.500
Age at onset	59 (20–86)	58 (34–72)	52 (19–76)	58.5 (55–62)	0.771	0.797
Disease duration	4 (0–20)	6 (1–31)	2 (0–21)	11 (3–19)	0.099	0.236
FARS stage	2 (1–4)	2.5 (2–4)	2 (1.5–4)	3 (3–3)	0.212	0.346
Use of walking aid	7 (8%)	2 (20%)	4 (29%)	0 (0%)	0.071	0.183
History of falls	15/59 (25%)	1/6 (17%)	2/7 (29%)	2 (100%)	0.166	0.303
Symptoms						
Episodic symptoms	29 (32%)	0 (0%)	6 (43%)	0 (0%)	0.066	0.183
Vertigo or dizziness	42 (47%)	1 (10%)	10 (71%)	0 (0%)	0.009	0.042
Oscillopsia	40/76 (53%)	4/7 (57%)	6/10 (60%)	1/1 (100%)	0.959	0.959
Gait unsteadiness	89/89 (100%)	10/10 (100%)	13/14 (93%)	2/2 (100%)	0.226	0.350
Fine motor problems	4/89 (4%)	2/8 (25%)	0/14 (0%)	1/2 (50%)	0.025	0.097
Speech problems	1/89 (1%)	2/9 (22%)	0/14 (0%)	1/2 (50%)	0.003	0.019
Sensory symptoms	15/89 (17%)	3/9 (33%)	3/14 (21%)	0/2 (0%)	0.575	0.615
Signs						
Impaired gait	68/88 (77%)	10/10 (100%)	11/14 (79%)	1/2 (50%)	0.211	0.346
Positive Romberg sign	37/82 (45%)	8/9 (89%)	7/13 (54%)	1/2 (50%)	0.060	0.183
Cerebellar impairment						
Cerebellar dysarthria	1/88 (1%)	3/9 (33%)	0/14 (0%)	1/2 (50%)	0.0007	0.011
Dysdiadochokinesia	7/88 (8%)	2/9 (22%)	0/14 (0%)	0/2 (0%)	0.306	0.431
Ataxia of upper limbs	5/88 (6%)	5/9 (56%)	2/14 (14%)	1/2 (50%)	0.0003	0.008
Downbeat nystagmus	7/90 (8%)	3/10 (30%)	2/14 (14%)	2/2 (100%)	0.002	0.017
Gaze-evoked nystagmus	35/88 (40%)	6/10 (60%)	4/14 (29%)	2/2 (100%)	0.145	0.281
Saccadic pursuit	41/88 (47%)	6/10 (60%)	6/14 (43%)	2/2 (100%)	0.500	0.574
Dysmetric saccades	8/88 (9%)	2/10 (20%)	2/14 (14%)	1/2 (50%)	0.127	0.271
Tremor of upper limbs	2 (2%)	1 (10%)	1 (7%)	0 (0%)	0.249	0.368
Parkinsonism	3 (3%)	2 (20%)	1 (7%)	0 (0%)	0.131	0.271
Impaired vibration at ankle	15/89 (17%)	7/10 (70%)	5/14 (36%)	0/2 (0%)	0.001	0.013
MRI	*n* = 58	*n* = 7	*n* = 7	*n* = 2		
Vermis atrophy	0 (0%)	2 (29%)	1 (14%)	0 (0%)	0.0095	0.042
Cerebellar hemisphere atrophy	1 (2%)	2 (29%)	1 (14%)	0 (0%)	0.031	0.107
Nerve conduction studies	*n* = 5/89	*n* = 5/10	*n* = 4/14	*n* = 0/2		
Axonal neuropathy	2/5 (40%)	3/3 (100%)	2/4 (50%)		0.356	0.477
Vestibular function						
VOR gain on vHIT	0.308 (0.005–0.575)	0.285 (0.05–0.35)	0.255 (0.165–0.405)	0.405 (0.22–0.59)	0.530	0.586

*FARS* Friedreich Ataxia Rating Scale; *vHIT* video head impulse test; *VOR* vestibulo-ocular reflex

Unless specified, data are reported as frequencies (percentages) for qualitative variables and median (range) for quantitative variables. Differences between groups were assessed with the non-parametric Kruskal–Wallis test for continuous variables and the Fisher’s exact test for categorical variables. *P* value of < 0.05 is considered significant, using the Benjamini–Hochberg method to correct for multiple comparisons. Data on age at onset were missing for seven patients in the “idiopathic” BVP and one patient in the *RFC1* positive group.

^a^The “idiopathic” BVP group includes two patients carrying non-pathogenic (GAAGGA)_n_ expansions

Cerebellar dysarthria and downbeat nystagmus were numerically more prevalent in patients with GAA-*FGF14*-related BVP compared to “idiopathic” BVP, although these differences did not maintain statistical significance after correcting for multiple comparisons (cerebellar dysarthria: 1/2, 50% vs 1/88, 1%; post-hoc pairwise Fisher test *p* = 0.044, Holm-adjusted *p* = 0.22; downbeat nystagmus: 2/2, 100% vs 7/90, 8%; post-hoc pairwise Fisher test *p* = 0.0086, Holm-adjusted *p* = 0.052). No other clinical features differed significantly between GAA-*FGF14*-related BVP and the other groups. However, the small size of the GAA-*FGF14*-related BVP group limited statistical power to detect differences.

## Discussion

In this study, we systematically screened a large, well-phenotyped consecutive cohort of patients with definite BVP for *FGF14* GAA repeat expansions. Our findings suggest that the phenotypic spectrum of GAA-*FGF14* disease can indeed include a relevant vestibular component (BVP); however*, FGF14* GAA expansions are overall a rare cause of primary BVP syndromes and are not observed in individuals with isolated BVP, occurring only in those with additional cerebellar signs, in particular DBN. These findings suggest that, while GAA-*FGF14* disease should be considered in the differential diagnosis of BVP, it represents a rare monogenic cause when BVP is the predominant presenting feature.

While vestibular dysfunction was recognized early as part of the phenotypic spectrum of GAA-*FGF14* disease [15], a recent study employing quantitative vestibular testing confirmed that it is a core feature of the disease and likely reflects central vestibulo-cerebellar involvement [13]. Importantly, however, BVP in GAA-*FGF14* disease typically emerges later in the disease course and appears secondary to cerebellar dysfunction rather than being the initial or dominant clinical manifestation [10]. Consistent with this observation, both GAA-*FGF14*-positive patients in the “idiopathic” BVP group had clear cerebellar signs on examination, including DBN, and a phenotype otherwise typical of GAA-*FGF14* disease, supporting the notion that primary cerebellar involvement remains the hallmark of this disorder.

Taken together, these findings argue against *FGF14* GAA repeat expansions being a frequent cause of isolated or primary BVP. Instead, they support a spectrum model of GAA-*FGF14* disease in which vestibular dysfunction represents a secondary feature, developing over time in the context of cerebellar degeneration. This distinction is clinically relevant, as it suggests that systematic screening for *FGF14* expansions in unselected BVP cohorts is likely to have a low diagnostic yield, whereas targeted testing may be more appropriate in patients with BVP accompanied by cerebellar impairment.

Our study also highlights the importance of considering dual genetic diagnoses in patients with complex or atypical phenotypes. One patient with BVP from our series carried both biallelic *RFC1* expansions and a GAA-*FGF14* expansion. This finding supports recent work demonstrating that *FGF14* expansions may co-occur with other pathogenic variants and modulate disease onset, progression, and phenotypic expression [9, 23–25]. In fact, patients carrying both a GAA-*FGF14* expansion and biallelic *RFC1* expansions have now been identified in a number of studies [23–25]. Together, these findings indicate that co-occurrence of GAA-*FGF14* and *RFC1* repeat expansions is possible and may occur more frequently than previously appreciated, as suggested by the relatively high population frequency of these expanded alleles [7, 17, 21, 26]. Dual diagnosis should thus be actively looked for in patients with unusual or broader phenotypes; there should be a low threshold to pursue additional genetic testing when clinical features are not fully explained by a single diagnosis, as co-occurring pathogenic variants may have implications for counseling.

Recent studies have shown that alleles in the intermediate range (200–249 GAA repeats) may be pathogenic, as suggested by their enrichment in patients with DBN and/or ataxia, although they remain challenging to interpret due to their incomplete penetrance [10, 17, 20, 21]. In our cohort, the four individuals carrying such alleles did not exhibit DBN or cerebellar ataxia, which are key features of GAA-*FGF14* disease, arguing against a clear contributory role in the BVP phenotype observed here. These findings support a cautious interpretation of intermediate alleles and reinforce the need for additional clinical and, ideally, familial evidence before attributing causality.

Several limitations of our study should be acknowledged. The small number of GAA-*FGF14*-positive patients limits statistical power and precludes definitive conclusions regarding discriminative clinical features. The incomplete availability of clinical data and ancillary investigations, including brain MRI, may have led to under-recognition of certain phenotypic features. In addition, this was a retrospective study conducted at a single tertiary referral center specializing in vestibular and balance disorders, which may introduce referral bias and limit generalizability to broader BVP populations. The retrospective design also precluded standardized longitudinal follow-up and may have resulted in variability in the completeness of clinical and ancillary data across patients. Finally, although this cohort represents the largest deeply phenotyped BVP series studied to date, it was drawn from a single-centre cohort, and its retrospective design precluded assessment of phenotypic evolution from disease onset in GAA-*FGF14*-positive patients.

In conclusion, our results demonstrate that *FGF14* GAA expansions are a rare monogenic cause of primary BVP syndromes, and that the phenotypic spectrum of GAA-*FGF14* disease can include a relevant vestibular component (BVP). Given the possible co-occurrence of GAA-*FGF14* and *RFC1* expansions, dual diagnosis should be considered in patients presenting with unusual or broader phenotype.

## Data Availability

Individual deidentified patient data may be shared at the request of any qualified investigator upon reasonable request.
